# Effects of coated sodium butyrate on the growth performance, serum biochemistry, antioxidant capacity, intestinal morphology, and intestinal microbiota of broiler chickens

**DOI:** 10.3389/fmicb.2024.1368736

**Published:** 2024-04-08

**Authors:** Jinwang Hou, Lizhi Lu, Lina Lian, Yong Tian, Tao Zeng, Yanfen Ma, Sisi Li, Li Chen, Wenwu Xu, Tiantian Gu, Guoqin Li, Xin Liu

**Affiliations:** ^1^State Key Laboratory for Managing Biotic and Chemical Threats to the Quality and Safety of Agro-products, Institute of Animal Husbandry and Veterinary Medicine, Zhejiang Academy of Agricultural Sciences, Hangzhou, China; ^2^College of Standardization, China Jiliang University, Hangzhou, China

**Keywords:** coated sodium butyrate, growth performance, serum antioxidant, gut microbes, yellow-feathered broiler

## Abstract

**Introduction:**

This study examined the impact of adding coated sodium butyrate (CSB) to the diet on the growth performance, serum biochemistry, antioxidant capacity, intestinal morphology, and cecal microbiota of yellow-feathered broiler chickens.

**Methods:**

In this study, 240 yellow-feathered broiler chickens at 26 days old were divided into two groups: the control group (CON group) received a standard diet, and the experimental group (CSB group) received a diet with 0.5 g/kg of a supplement called CSB. Each group had 6 replicates, with 20 chickens in each replicate, and the experiment lasted for 36 days.

**Results:**

Compared to the CON group, the CSB group showed a slight but insignificant increase in average daily weight gain during the 26–62 day period, while feed intake significantly decreased. The CSB group exhibited significant increases in serum superoxide dismutase, catalase, and total antioxidant capacity. Additionally, the CSB group had significant increases in total protein and albumin content, as well as a significant decrease in blood ammonia levels. Compared to the CON group, the CSB group had significantly increased small intestine villus height and significantly decreased jejunal crypt depth. The abundance of *Bacteroidetes* and *Bacteroides* in the cecal microbiota of the CSB group was significantly higher than that of the CON group, while the abundance of *Proteobacteria*, *Deferribacteres*, and *Epsilonbacteraeota* was significantly lower than that of the CON group.

**Conclusion:**

These results suggest that adding CSB to the diet can improve the growth performance and antioxidant capacity of yellow-feathered broiler chickens while maintaining intestinal health.

## Introduction

As the standard of living continues to rise, people are increasingly concerned about the safety and health aspects of animal food products. Consequently, food safety has rapidly emerged as a major global concern (Luo et al., 2021). In the past, antibiotics were used as growth promoters to maintain animal health and improve the economic efficiency of farms (Roth et al., 2019). However, the negative impacts they bring are undeniable. Therefore, there is an urgent need for animal nutritionists to seek novel, environmentally friendly feed additives to meet the demands of sustainable livestock development (Barrett et al., 2021). The research and application of these novel feed additives have become a hot topic of investigation (Casewell et al., 2003). Selecting safe feed additives that promote health, sustainability, and environmental stewardship has become a major trend in China and worldwide. Furthermore, as the productivity of commercial broiler chickens continues to advance and the breeding cycle shortens, the rapid growth exacerbates the intestinal burden of broiler chickens, making them susceptible to oxidative stress (Mishra and Jha, 2019; Han et al., 2020). The healthy growth of broiler chickens is intricately entwined with their regular diet and intestinal well-being (Vancamelbeke and Vermeire, 2017; Amer et al., 2021). Research suggests that the health of the intestinal microbiota mirrors the digestive and absorptive capacity of the intestine (Jia et al., 2022). A balanced intestinal microbiota can promote the maturation and development of the host immune system, playing a crucial role in the overall health of the host (Fang et al., 2019).

Sodium butyrate, also known as butanoic acid, is primarily composed of butyric acid, a short-chain fatty acid (SCFAs) (Huang et al., 2023). Its main metabolic products include ketone bodies and CO_2_, which serve as a source of energy for intestinal epithelial cells (Hamer et al., 2008; Bedford and Gong, 2018). Furthermore, it has been demonstrated to diminish the occurrence of intestinal inflammation, thus playing a beneficial role in regulating gut health (Zhang et al., 2021). Sodium butyrate can enhance intestinal morphology, promote the differentiation of intestinal epithelial cells, maintain the balance of intestinal microbiota, inhibit the growth of harmful bacteria, and increase the proliferation of beneficial bacteria (Liu et al., 2020; El-Saadony et al., 2022; Kaźmierczak-Siedlecka et al., 2022). Consequently, it augments the digestion and absorption rates of nutrients, effectively improving the growth performance of animals (Ficagna et al., 2022). However, the foul odor and irritating taste of sodium butyrate not only have a negative impact on animal intake, but also readily absorb moisture when exposed to air, leading to significant detrimental effects on feed production and storage, ultimately severely compromising animal palatability (Le Gall et al., 2009). Coated sodium butyrate (CSB) can effectively address this drawback by controlling the odor and enabling a slow release of sodium butyrate in the animal intestines, thereby maximizing its efficacy (Sun et al., 2022; Miao et al., 2023). CSB has no negative effect on feed intake and could increase feed efficiency (Bloomer et al., 2019). Therefore, most of the sodium butyrate products currently used in practice are coated with fat or starch to reduce the irritating odor of sodium butyrate itself and provide slow release in the intestine, resulting in a more stable effect compared to uncoated sodium butyrate (Lin et al., 2020; Zhang et al., 2022).

The yellow-feathered broilers, known for their delicious meat, lack research on the use of CSB as a feed additive. We hypothesize that adding CSB to their diet improves their growth performance, serum biochemistry, antioxidant capacity, and gut health. To test this hypothesis, we investigated the effects of CSB on these factors in yellow-feathered broiler chickens. The study aims to establish a theoretical basis for the use of CSB in the production of yellow-feathered broiler chickens, known for their highly valued meat.

## Materials and methods

### Materials

The CSB used in the experiment contains a minimum of 30% sodium butyrate, and was provided by Hangzhou Kangdequan Feed Co., Ltd. (Hangzhou, Zhejiang Province, China).

### Animals, diets, and experimental design

The experiment selected yellow-feathered broilers (Hexi dwarf female broilers) with medium growth rate as the experimental animals. The growth cycle of this variety spans 62 days and is divided into three rearing stages: the brooding period (1 to 25 days), the growing period (26 to 40 days) and the fattening period (41 to 62 days). The experimental subjects chosen for this study were yellow-feathered broilers aged 26–62 days.

Two hundred forty yellow-feathered broilers were randomly assigned into two groups, with 6 replicates and 20 broilers per replicate in each group. The control group was fed a basal diet (CON group), while the experimental group had CSB with 0.5 g/kg added to the basal diet (CSB group). Prior to the commencement of the experiment, a comprehensive cleaning and disinfection of the poultry house was undertaken, and individual enclosures were established for each replicate. All experimental animals were maintained under identical husbandry conditions, with continuous illumination provided for 24 h per day. The birds were reared on the ground (with a 4–5 cm layer of rice husks) and were fed and water troughs cleaned at 8 am and 5 pm each day. The experimental site and test animals were provided by Qiling Agriculture and Animal Husbandry Co., Ltd. in Yudu County, Ganzhou City, Jiangxi Province. This study has been approved by the Experimental Animal Ethics Committee of Zhejiang Academy of Agricultural Sciences (Hangzhou, China). The basal diet was formulated based on the Agricultural Industry Standard of the People’s Republic of China - Chicken Feed (NY_T33-2004) and modified in accordance with production practices. The composition and nutritional components of the feed provided are shown in Table 1 (in granular form).

**Table 1:** Composition and nutrient levels of the basal diet (as-dry basis, %).

Ingredients, %	Content		Nutrient levels	Content	
	26 to 40 days	41 to 62 days		26 to 40 days	41 to 62 days
Wheat	77.96	81.51	ME ^3^, MJ/kg	12.76	12.97
Soybean meal 43%	5.65	0.00	Crude protein, %	17.50	16.50
Sunflower meal	3.50	4.50	Crude fat, %	4.76	5.17
Peanut meal	4.00	4.25	Crude fiber, %	2.39	2.31
Corn gluten meal 60%	1.56	1.28	Calcium, %	0.80	0.75
Feather meal	0.00	1.00	Sodium, %	0.19	0.20
CaHPO4	0.63	0.47	Available phosphorus, %	0.30	0.28
Limestone	1.36	1.33	Lysine, %	0.90	0.85
Soybean oil	3.14	3.46	Methionine, %	0.45	0.37
Premix	2.20 ^1^	2.20 ^2^			
Total	100.00	100.00			

1 Premix from 26 to 40 days of age was provided per kilogram of diets: NaHCO3 2 g, NaCl 2 g, Methionine 2.17 g, Threonine 1.73 g, Lysine sulphate 7.95 g, VA 10,000 IU, VD3 3,000 IU, VE 30 mg, VK3 1.3 mg, VB6 4 mg, VB12 0.013 mg, Thiamine 2.2 mg, Riboflavin 8 mg, Nicotinamide 40 mg, Choline chloride 600 mg, Calcium pantothenate 10 mg, Biotin 0.04 mg, Folic acid 1 mg, Fe 80 mg, Cu 7.5 mg, Mn 110 mg, Zn 65 mg, I 1.1 mg, Se 0.3 mg.

2 Premix from 41 to 62 days of age was provided per kilogram of diets: Methionine 1.50 g, Threonine 1.97 g, Lysine sulphate 8.93 g, The rest of the ingredients are the same as those of the 26- to 40-day-old premix.

3 ME = metabolizable energy.

### Sample collection

At 62 days of age, following a 12-h fasting period, one experimental broiler chicken with a body weight close to the group mean was selected from each replicate (comprising 6 individuals) for sample collection. Blood samples were collected via the wing vein and then centrifuged at 3000 rpm for 10 min at 4°C for the determination of serum indexes. Additionally, tissue samples (2–3 cm segments of the duodenum, jejunum, and ileum) were collected from each selected experimental animal and placed in a 4% paraformaldehyde solution for intestinal morphology assessment. Finally, cecal contents were collected, transferred to 2 mL cryotubes, flash-frozen in liquid nitrogen, and stored at −80°C for subsequent 16S rRNA gut microbiota analysis.

### Growth performance

During the experiment, the health and mental state of the chickens were observed daily (by observing whether they have agile movements, strong appetite, and sensitivity to external stimuli to determine if their mental state is normal), and the daily feed intake was recorded for each replicate. The fasted body weights of the chickens were measured and recorded at 26, 40, and 62 days of age, after a fasting period of 12 h, for the purpose of calculating the average daily gain (ADG), average daily feed intake (ADFI), and feed conversion ratio (FCR) during the 26–40 day and 41–62 day periods.

### Serum antioxidant analysis

The serum antioxidant indicators comprise the measurement of total antioxidant capacity (T-AOC), malondialdehyde content (MDA), catalase (CAT), superoxide dismutase (SOD), and glutathione peroxidase (GSH-PX), with the respective kit catalog numbers: HY-60021, HY-M0003, HY-M0018, HY-M0001, and HY-M0004. All kits are supplied by the Beijing Huaying Biotechnology Research Institute, and are rigorously operated in accordance with the actual kit instructions.

### Serum biochemical analysis

The serum biochemical markers include: total protein (TP), albumin (ALB), total cholesterol (TC), triglycerides (TG), alkaline phosphatase (ALP), blood ammonia (AN), low-density lipoprotein (LDL), and high-density lipoprotein (HDL), with the respective kit catalog numbers: HY-50067, HY-50068, HY-50061, HY-50062, HY-N0005, HY-N0047, HY-50071, and HY-50070. All kits are provided by the Beijing Huaying Biotechnology Research Institute, and are strictly operated in accordance with the actual kit instructions.

### Small intestinal histomorphology analysis

Tissue samples of the duodenum, jejunum, and ileum, each measuring 2–3 cm, were obtained from various parts of the small intestine. These samples are then fixed in a 4% paraformaldehyde solution for a minimum of 72 h, followed by dehydration, washing, paraffin embedding, sectioning, and staining with hematoxylin–eosin. Images are captured at 40x magnification using an optical microscope (Eclipse Ci-L, Nikon, Japan), with a focus on clear areas of interest. Analysis is conducted using ImagePro Plus 6.0 software (Media Cybernetics, Bethesda, MD, USA), with measurement of villus height (VH) and crypt depth (CD) at 15 different locations within each group, and subsequent calculation of the villus height to crypt depth ratio (V/C).

### Microbial 16S rRNA analysis of Cecal Digesta

The cecal chyme samples stored in the −80°C freezer were retrieved for the determination of intestinal microbial diversity indices. Following the manufacturer’s guidelines, microbial DNA was extracted from the cecal contents using the QIAamp DNA Stool Mini Kit (QIAGEN, CA, Hamburg, Germany). Subsequently, markers and adaptor-linked universal primers 341F (5′-CCTAYGGRBGCASCAG-3′) and 806R (5′-GGACTACNNGGGTATCTAAT-3′) targeting the V3-V4 region were employed to amplify microbial 16S rRNA. The amplification of the 16S rRNA gene via PCR proceeded as follows: all PCR reactions were conducted in a 30 μL reaction mix, comprising 15 μL of Phusion^@^High-Fidelity PCR Master Mix (New England Biolabs), 0.2 μM each of forward and reverse primers, and approximately 10 ng of template DNA. The thermal cycling entailed an initial denaturation at 98°C for 1 min, followed by denaturation at 98°C for 30 s, annealing at 50°C for 30 s, extension at 72°C for 60 s, and a final extension at 72°C for 5 min. The purity of the PCR products was assessed using 2% agarose gel, and the products were purified using the GeneJET DNA Gel Extraction Kit (Thermo Fisher Scientific, Waltham, MA, United States). The DNA library was established using the TruSeq DNA PCR-Free Sample Preparation Kit, followed by quantification and library assessment using Qubit. Sequencing was performed on the Illumina Novaseq 6,000 platform (Illumina, San Diego, CA, USA). Data from different samples were distinguished based on barcode sequences, and the extracted data were saved in fastq format. For paired-end (PE) data, each sample yielded two files (fq1 and fq2), representing reads from the two ends of the sequencing. Subsequently, the FLASH software^1^ was employed for the assembly of paired-end sequences. Quality control filtering was applied to the reads, as well as to evaluate the merging efficiency, resulting in the acquisition of clean data. All samples were processed using the Uparse software (Uparse v7.0.1001) for operational taxonomic unit (OTU) clustering at a 97% similarity threshold, followed by taxonomic annotation of the representative sequences of the OTUs using the Greengenes database.^2^

### Statistical analysis

Statistical analysis was conducted using the SPSS 29.0 software (SPSS; IBM Corp, Armonk, NY, United States). Phenotypic analysis was conducted using the Shapiro–Wilk test, and for the analysis of differences under the assumption of normal distribution, Student’s *t*-test was employed. Alpha diversity was assessed using the Observed species, Chao1, Ace, Shannon, and Simpson indices. Beta diversity was evaluated based on the weighted UniFrac distance matrix and visualized using Principal Coordinate Analysis (PCoA). Both alpha and beta diversities were calculated for significant differences using a *t*-test ANOVA. In the LEfSe analysis, non-parametric Kruskal-Wallis rank sum tests were used to identify species with significant differences in abundance between groups, followed by paired Wilcoxon rank sum tests. The linear discriminant analysis(LDA) was used to estimate the effect size of each differentially abundant feature, and the threshold on the LDA score (log10 LDA) was set to 2.0.

## Results

### Growth performance

**Figure 1:**
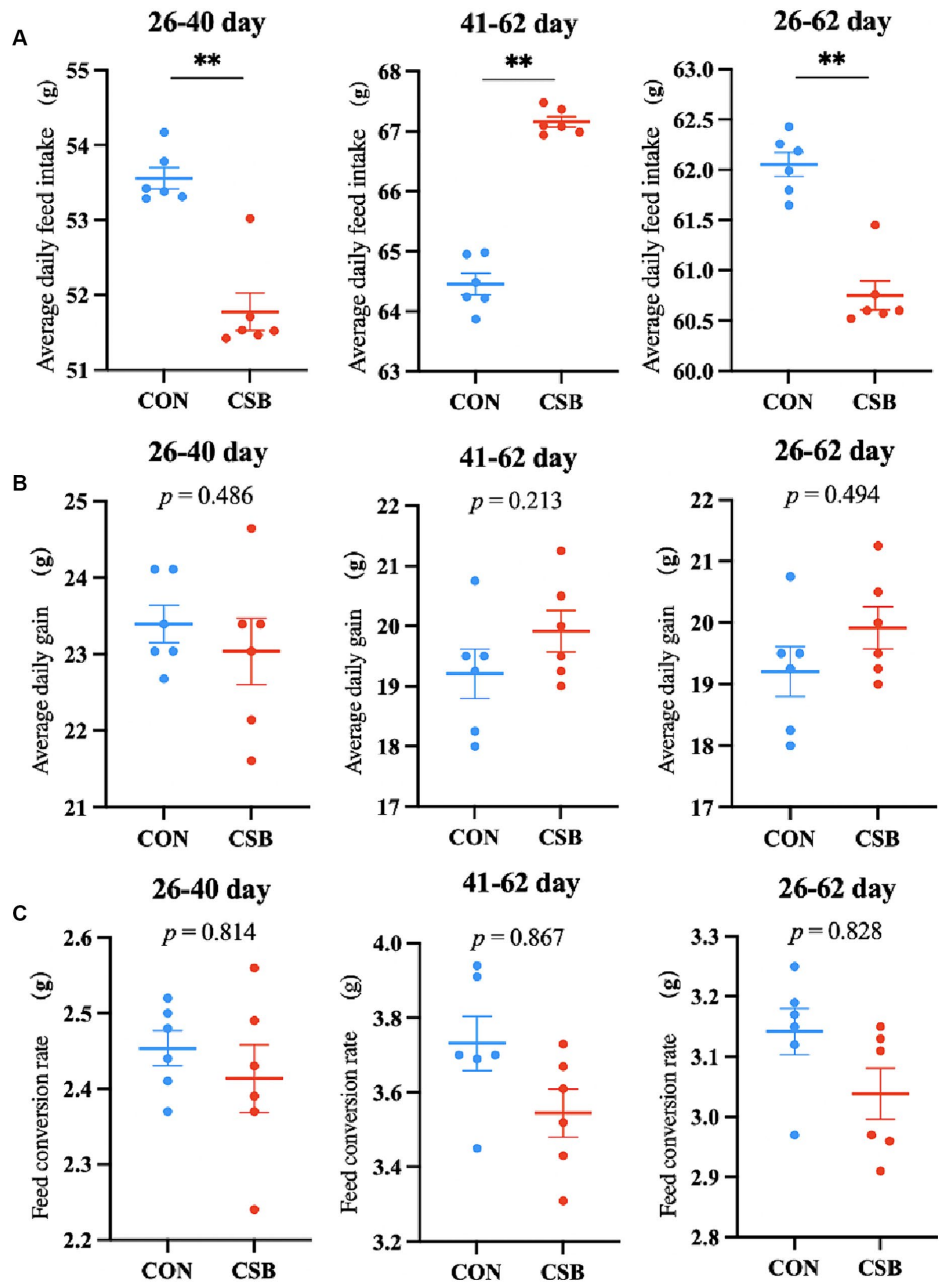
The effect of adding CSB to the feed on the growth performance of broiler chickens with yellow feathers. **(A)** Average daily feed intake (ADFI). **(B)** Average daily gain (ADG). **(C)** Feed conversion rate (FCR). Values are presented as means ± SEM (*n* = 6). **p < 0.01. CON, control group (fed with basic diet); CSB group, in which CSB (0.5 g/kg) was added to the basic feed. Method: *T*-test.

The impact of CSB on the growth performance of broiler chickens is illustrated in Figure 1. In comparison with the control group, the addition of CSB significantly reduced ADFI at the ages of 26–40 days, 41–62 days, and 26–62 days (*p* < 0.01); no statistical significance was observed in ADG and FCR at each stage (*p* > 0.05).

### Serum antioxidant analysis

**Figure 2:**
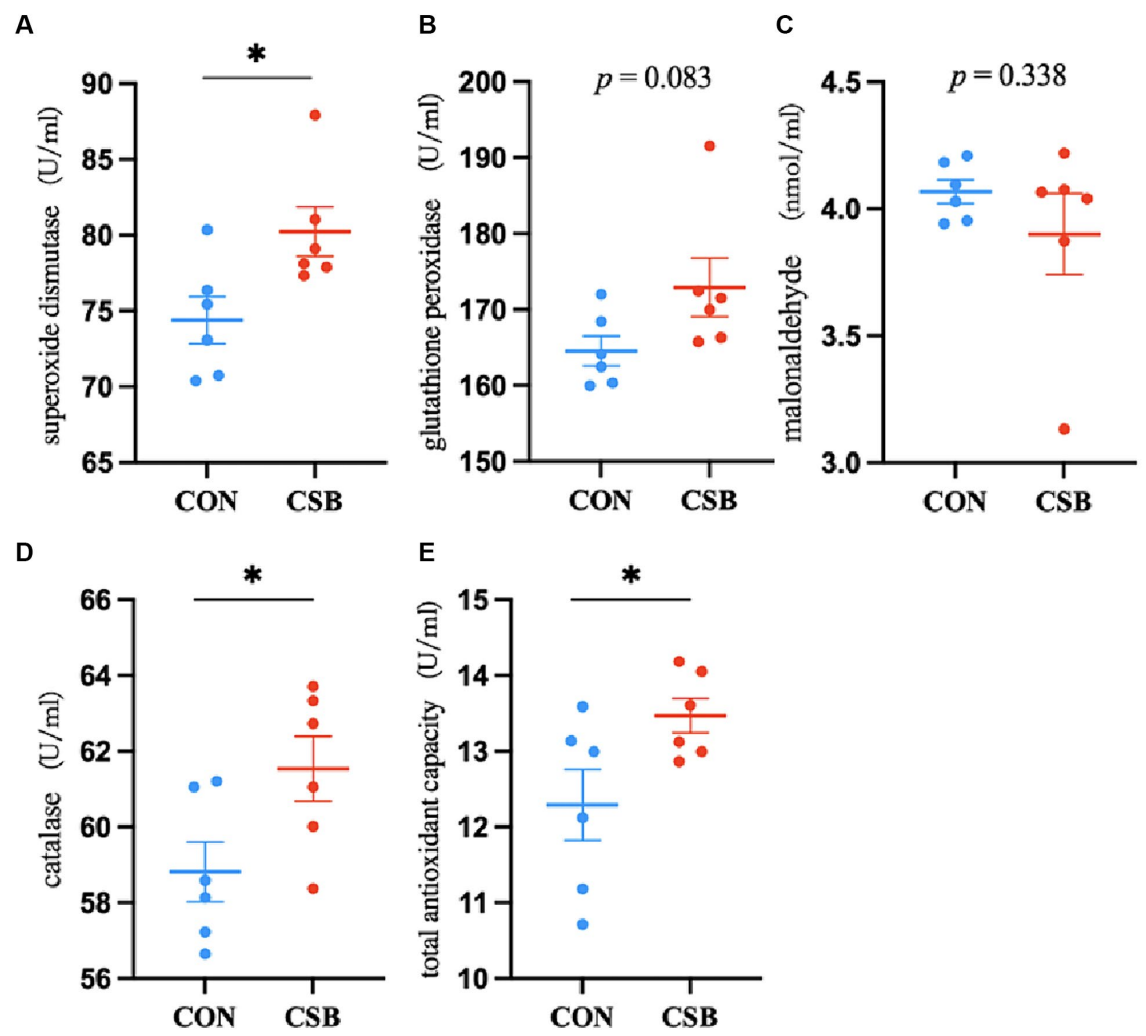
The effect of adding CSB to the feed on the serum antioxidant and immune parameters of broiler chickens with yellow feathers. **(A)** Superoxide dismutase(SOD); **(B)** glutathione peroxidase(GSH-Px); **(C)** malonaldehyde(MDA); **(D)** catalase(CAT); **(E)** total antioxidant capacity(T-AOC). Values are presented as means ± SEM (*n* = 6). CON, control group (fed with basic diet); CSB group, in which CSB (0.5 g/kg) was added to the basic feed. Method: *T*-test.

The impact of CSB on the serum antioxidant indicators of broiler chickens is depicted in Figure 2. In comparison with the control group, the addition of CSB significantly increased the levels of SOD, CAT, and T-AOC (*p* < 0.05), while no statistical significance was noted in GSH-Px and MDA levels (*p* > 0.05).

### Serum biochemical analysis

**Figure 3:**
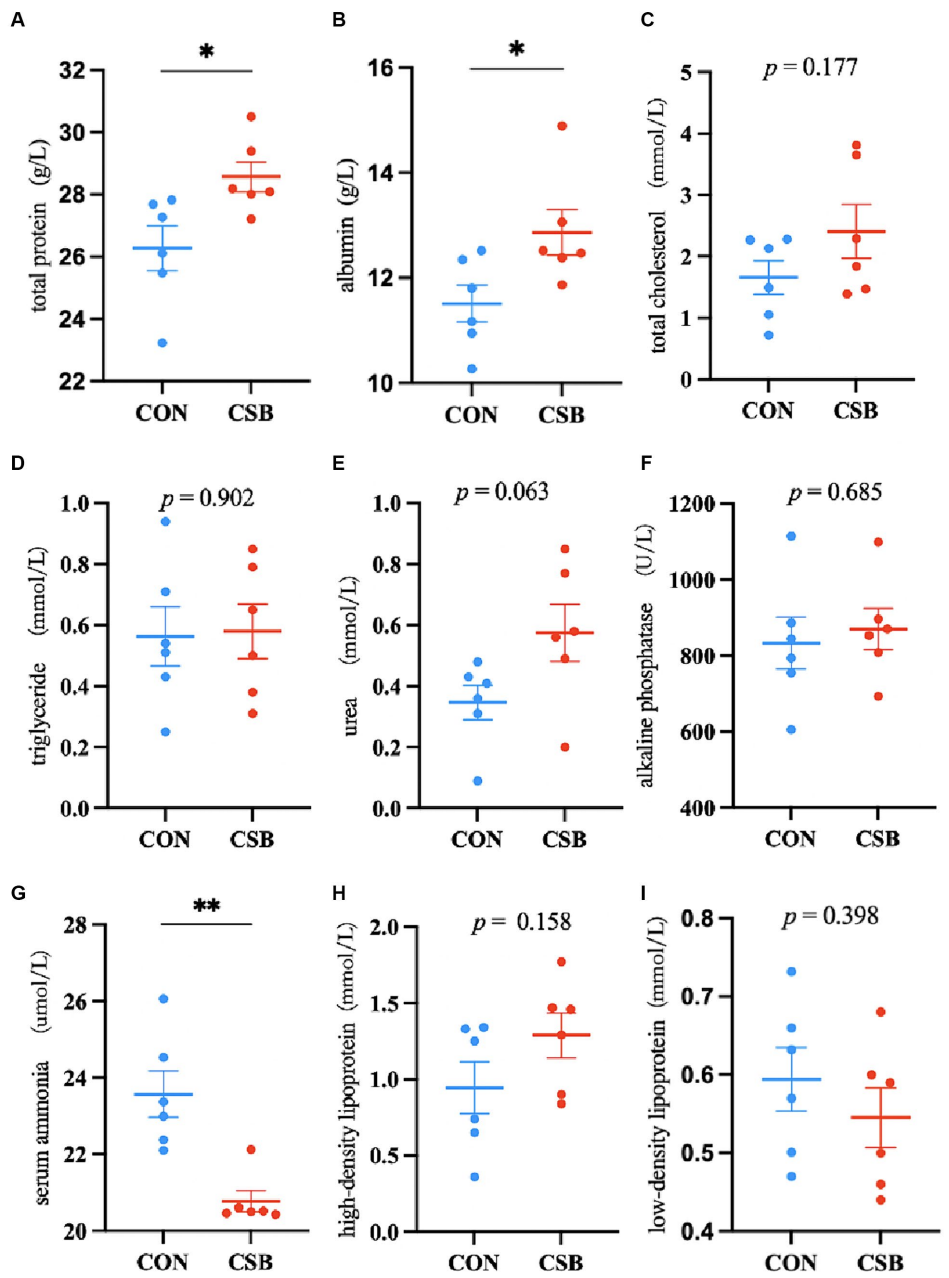
The effect of adding CSB to the feed on the serum biochemical parameters of broiler chickens with yellow feathers. **(A)** total protein(TP); **(B)** albumin(ALB); **(C)** total cholesterol(TC); **(D)** triglyceride (TG); **(E)** Urea(UREA); **(F)** alkaline phosphatase(ALP); **(G)** serum ammonia(AN); **(H)** high-density lipoprotein(HDL); **(I)** low-density lipoprotein(LDL). Values are presented as means ± SEM (*n* = 6). *p < 0.05. **p < 0.01. CON, control group (fed with basic diet); CSB group, in which CSB (0.5 g/kg) was added to the basic feed. Method: T-test.

The impact of CSB on the serum biochemical indicators of broiler chickens is illustrated in Figure 3. Compared to the control group, the addition of CSB resulted in a significant decrease of 11.88% in serum AN levels (*p* < 0.01), as well as significant increases of 8.76 and 11.77% in TP and ALB levels, respectively (*p* < 0.05), while no statistical significance was observed in TC, TG, UREA, ALP, HDL, and LDL levels (*p* > 0.05).

### Small intestinal Histomorphology analysis

**Figure 4:**
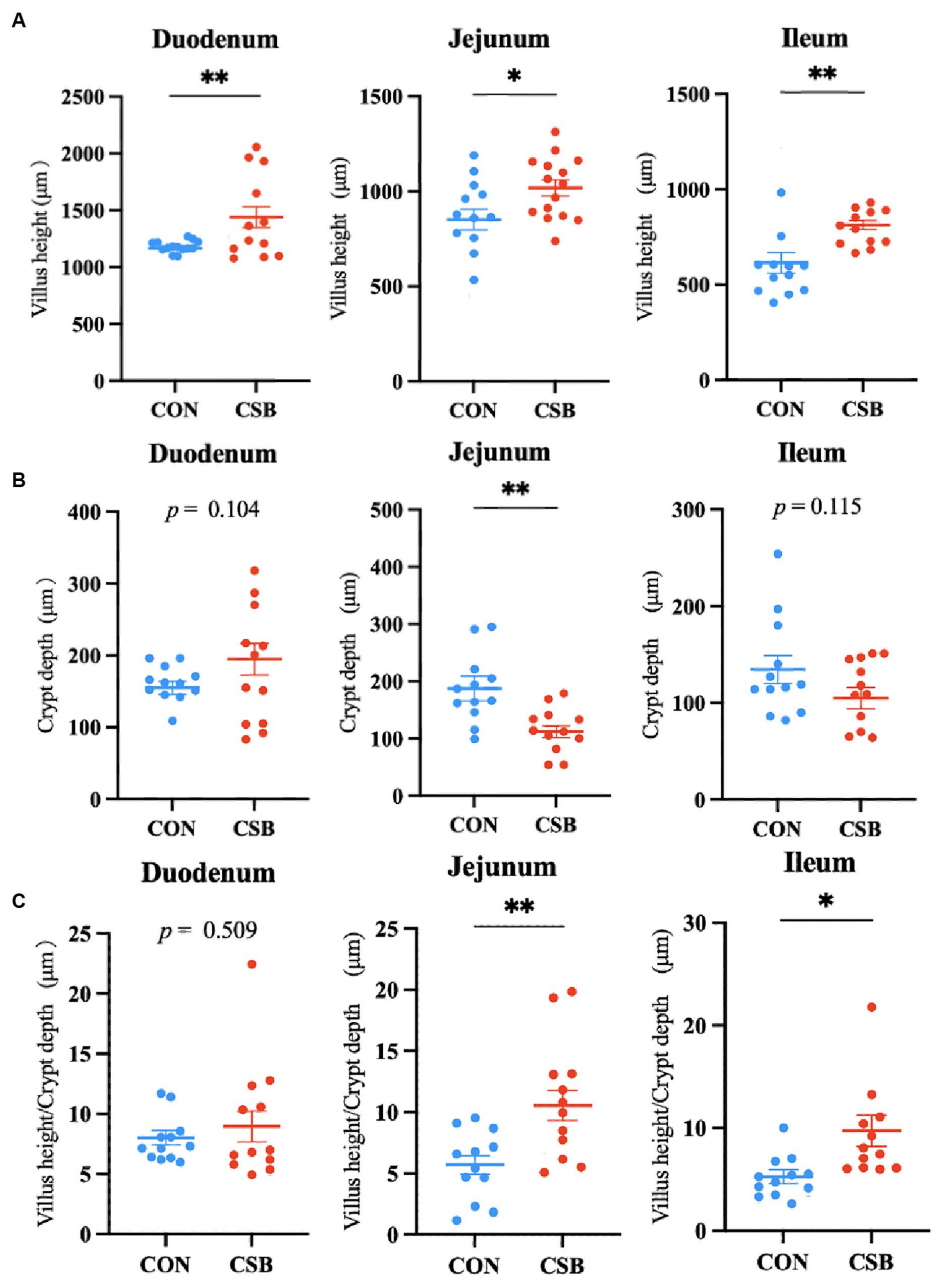
The effect of adding CSB to the feed on the intestinal morphology of broiler chickens with yellow feathers. **(A)** Villus height(VH); **(B)** crypt depth (CD); **(C)** villus height/Crypt depth(V/C). Values are presented as means ± SEM (*n* = 6). *p < 0.05. **p < 0.01. CON, control group (fed with basic diet); CSB group, in which CSB (0.5 g/kg) was added to the basic feed. Method: T-test.

**Figure 5:**
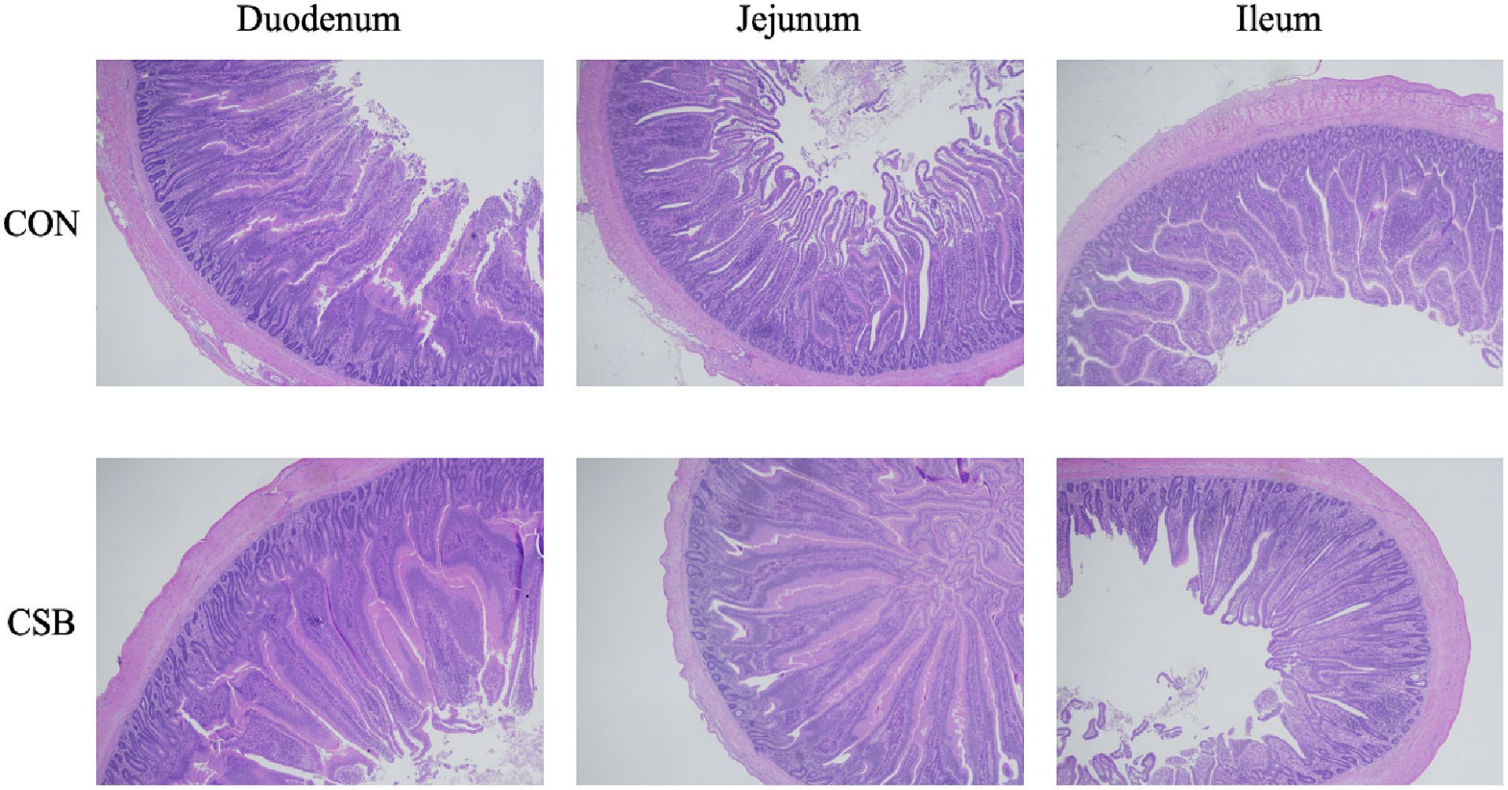
Microscopic images of the small intestine (scale bar = 500 μm). CON, control group (fed with basic diet); CSB group, in which CSB (0.5 g/kg) was added to the basic feed.

The impact of CSB on the intestinal morphology of yellow-feathered broiler chickens is illustrated in Figure 4. Compared to the control group, the addition of CSB resulted in a 23.39% increase in the VH value of the duodenum and a 19.48% increase in the VH value of the jejunum (*p* < 0.01). In addition, compared to the control group, the CSB group showed a 32.2% increase in VH value in the ileum (*p* < 0.05), a 21.64% decrease in CD value (*p* < 0.05), and a significant improvement in V/C value in the ileum and jejunum (*p* < 0.05). The microscopic images of the two intestinal tracts are depicted in Figure 5.

### Microbial 16S rRNA analysis of Cecal Digesta

**Figure 6:**
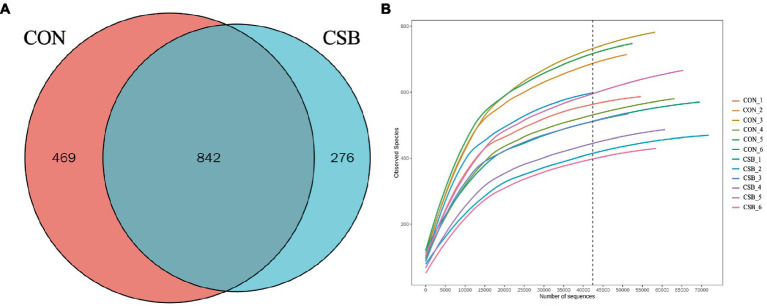
**(A)** Analysis of the operational taxonomic units (OTUs) shared among groups. Each circle in the figure indicates a group, and the numbers in the overlapping circles indicate the number of OTUs shared between groups. Numbers located in non-overlapping areas indicate the number of unique OTUs in the group. **(B)** Rarefaction curve. CON, control group (fed with basic diet); CSB group, in which CSB (0.5 g/kg) was added to the basic feed.

**Figure 7:**
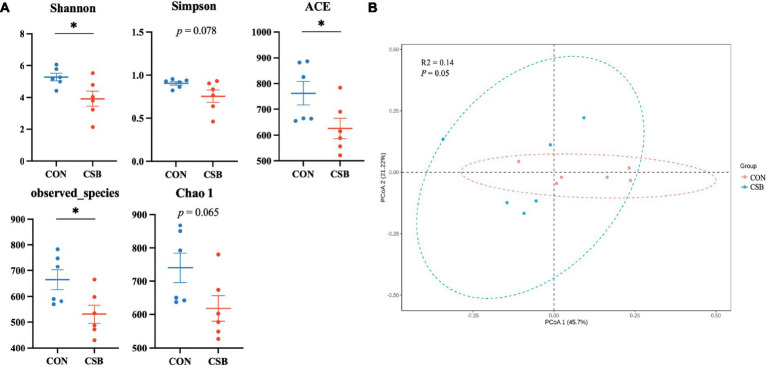
**(A)** The effect of adding CSB to the feed on the cecal microbial alpha diversity of broiler chickens with yellow feathers. The number of Observed species corresponds to the number of operational taxonomic units (OTUs); Shannon and Simpson indices represent the microbial community diversity; Chao 1 and ACE indices indicate the microbial community abundance. **(B)** Principal coordinate analysis (PCoA) of the cecal microbiota based on weighted UniFrac distances. PCoA1, first principal coordinate; PCoA2, second principal coordinate. Values are presented as means ± SEM (*n* = 6). *p < 0.05. CON, control group (fed with basic diet); CSB group, in which CSB (0.5 g/kg) was added to the basic feed. Method: *t*-test.

In this study, a total of 1,587 operational taxonomic units (OTUs) were observed. The CON and CSB groups shared 842 OTUs, while the CON group had 469 unique OTUs, and the CSB group had 276 unique OTUs (Figure 6A). The rarefaction curve in Figure 6B indicates the sequencing data volume and species abundance. As depicted in Figure 7A, the Shannon, Simpson, Chao1, Observed species, and ACE indices were utilized to represent alpha diversity. In comparison with the CON group, the CSB group significantly reduced the Shannon, Observed species, and ACE indices (*p* < 0.05), with no statistical significance observed for Simpson and Chao1 (*p* > 0.05). Principal Coordinates Analysis (PCoA) was employed to investigate the similarity or dissimilarity of sample community composition. As shown in Figure 7B, the eigenvalues for PCoA1 and PCoA2 are 45.7 and 21.22%, respectively (*R*^2^ = 0.14, *p* = 0.05). There are noticeable differences in the microbial communities between the CON group and the CSB group, indicating a substantial impact of CSB on the cecal microbial community structure of yellow-feathered broiler chickens.

**Figure 8:**
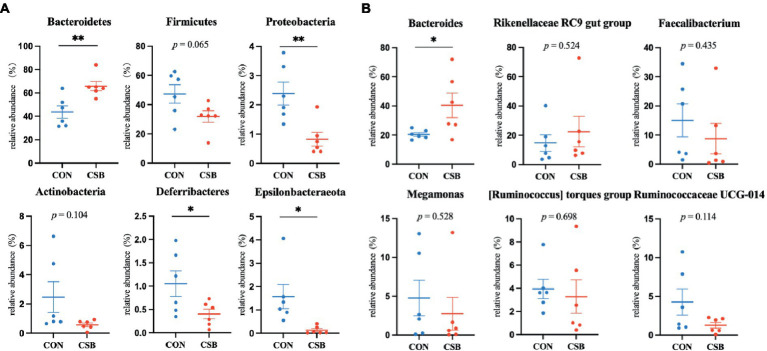
**(A)** The effect of CSBfeed on the microbial phylum-level composition in the ceca of broiler chickens. **(B)** The effect of CSB feed on the microbial genus-level composition in the ceca of broiler chickens. Values are presented as means ± SEM (*n* = 6). *p < 0.05. **p < 0.01. CON, control group (fed with basic diet); CSB group, in which CSB (0.5 g/kg) was added to the basic feed. Method: *t*-test.

The abundance of microbial communities in the cecum of the two animal groups was analyzed at the phylum and genus levels. From Figure 8A, it is apparent that at the phylum level, *Bacteroidetes*, *Firmicutes*, *Proteobacteria*, *Actinobacteria*, *Deferribacteres*, and *Epsilonbacteraeota* are the predominant taxa in the cecum. Specifically, the abundance of *Firmicutes* (47.30%) was observed to be the highest in the CON group, while *Bacteroidetes* (70.82%) exhibited the highest abundance in the CSB group. In comparison to the CON group, the CSB group displayed a significant increase in the abundance of *Bacteroidetes* (*p* < 0.01) and a notable decrease in the abundance of *Proteobacteria*, *Deferribacteres*, and *Epsilonbacteraeota* (*p* < 0.05), with no significant impact observed on other phylum-level microbial communities. Additionally, from Figure 8B, at the genus level, *Bacteroides*, *Rikenellaceae RC9 gut group*, *Faecalibacterium*, *Megamonas*, [*Ruminococcus*] *torques group*, and *Ruminococcaceae UCG-014* were identified as the major dominant taxa in the cecum. Notably, *Bacteroides* emerged as the most dominant taxa in both groups (20.41 and 40.45%). Furthermore, the addition CSB was observed to significantly increase the abundance of *Bacteroides* in the cecum of broiler chickens (*p* < 0.05), with no significant impact on other genus-level microbial communities.

**Figure 9:**
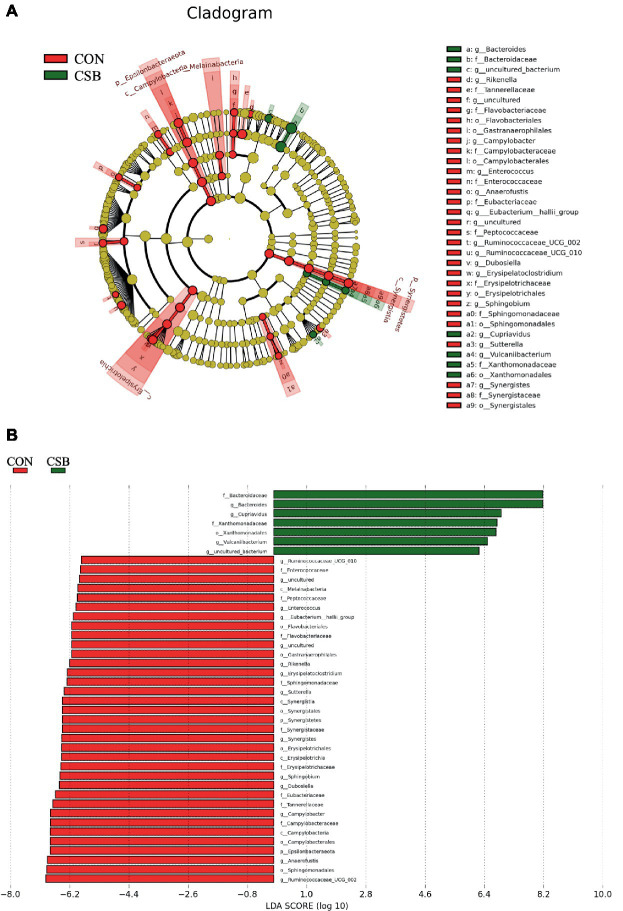
Linear discriminant analysis (LDA) effect size (LEfSe) was used to identify the most differentially abundant taxa in the cecal microbiota of the CON and CSB groups. **(A)** In the evolutionary branching diagram, the concentric circles radiating from inside to outside represent the taxonomic levels from phylum to genus (or species). Each small circle on different taxonomic levels represents a classification at that level, and the diameter of the small circle is directly proportional to its relative abundance. The red and green areas indicate different groups, with red nodes in the branches representing microbial taxa that play an important role in the CON group, green nodes representing microbial taxa that are significant in the CSB group, and yellow nodes representing microbial taxa that are not significant in either group. **(B)** In the LDA value distribution histogram, the red and green areas represent different groups, with the red area indicating microbial taxa that are significant in the CON group and the green area indicating those significant in the CSB group. Only species with an LDA Score > 2 are displayed in the diagram, and the length of the bars represents the magnitude of the LDA values. Method: Kruskal-Wallis rank sum test.

We conducted LefSe analysis to elucidate the differences in microbial abundance between the CON and CSB groups. As depicted in Figures 9A,B, a total of 42 branch taxa exhibited significant differences in abundance (LDA Score > 2; *p* < 0.05). Among these, 35 branch taxa were found to be significantly influenced in the CON group, including *f_Eubacteriaceae*, *f_Tannereilaceae*, *g_Cmpylobacter*, *f_Campylobacteraceae*, *c_Campylobacteria*, *o_Campylbbacterales*, *p_Epsilonbacteraeota*, *g_Anaerofustis*, *o_Sphingomonadales*, *g_Ruminococcaceae_UCG_002*, and others. Conversely, the CSB group exhibited significant impacts on 7 branch taxa, including *f_Bactergidaceae*, *g_Bacteroides*, *g_Cupriavidus*, *f_Xanthomonadaceae*, *o_Xanthomonadales*, *g_Vulcaniibacterium*, and *g_uncultured bacterium*.

## Discussion

The production performance serves as a crucial indicator reflecting the benefit of the animal husbandry industry. In this study, we investigated the effects of CSB as a feed additive on the growth performance of yellow-feathered broiler chickens. Our findings are in line with previous studies. Zeng et al. (2023), reported a slight reduction in ADFI in ducks with the addition of CSB, confirming our results. Additionally, Zhang et al. (2022), demonstrated that the inclusion of 800 mg/kg CSB in the feed significantly improved FCR, with no significant effect on ADFI. Consistently, Makowski et al. (2022), observed a reduction in feed conversion ratio in turkeys fed with CSB. Our experiment showed a significant reduction in ADFI in yellow-feathered broilers with the addition of CSB, while there were no significant changes in ADG and FCR between the CON and CSB groups. The improved nutrient absorption in the intestines due to CSB inclusion may explain the reduction in ADFI, indicating that a smaller amount of feed can fulfill the nutritional needs of broilers (Leonel and Alvarez-Leite, 2012). This study provides evidence that CSB can effectively enhance the growth performance of broiler chickens, reduce breeding costs, and potentially optimize production efficiency and economic sustainability in the poultry industry, thereby highlighting the potential of incorporating CSB in poultry feed.

Oxidative reactions occur in the bodies of animals and, while providing energy, give rise to harmful reactive oxygen species (ROS). The continuous accumulation of these detrimental ROS can lead to oxidative stress in livestock and poultry, adversely affecting their health (Kamboh et al., 2016). To counteract the continuous accumulation of free radicals, various antioxidant enzymes, such as superoxide dismutase (SOD) and catalase (CAT), play a significant role. Their activity maintains a stable equilibrium between ROS generation and elimination in the bodies of livestock and poultry. SOD is a metalloenzyme (Zhang et al., 2023), T-AOC and GSH-Px are comprehensive indicators of antioxidant substances and capacity in livestock and poultry (Qin et al., 2022), CAT is an enzyme that decomposes hydrogen peroxide into oxygen and water (Baker et al., 2023), and they all play important roles in eliminating ROS in the body, thereby improving the antioxidant capacity of livestock and poultry (Li et al., 2021). MDA activities are often used as a marker of free radicals-induced lipid peroxidation and as an indicator of oxidative damage (Zhou et al., 2006). Research by Miao et al. (2023), demonstrated that the addition of CSB to feed effectively increases the content of GSH-Px and T-AOC, while reducing MDA levels. Additionally, the research conducted by Zeng et al. (2023), also indicates that the addition of CSB can effectively enhance the serum antioxidant capacity of laying ducks. In line with these findings, our study revealed that the addition of CSB significantly increased the levels of SOD, CAT, and T-AOC in the serum of broiler chickens, providing compelling evidence for the beneficial effects of CSB in enhancing the serum antioxidant capacity of yellow-feathered broilers.

The biochemical parameters of serum in broiler chickens serve as crucial indicators for evaluating their health status and productivity, reflecting the metabolic and physiological functions within the avian body (Zhang et al., 2018; Omar et al., 2021). TP and ALB represent pivotal markers for protein metabolism, offering comprehensive insights into the synthesis and degradation equilibrium of proteins within broiler chickens (Chen et al., 2014). In our investigation, the inclusion of CSB in the diet of yellow-feathered broiler chickens markedly elevated the levels of TP and ALB. In a similar vein, the study by Bawish et al. (2023), also indicated a significant increase in TP and ALB levels with the addition of sodium butyrate to broiler feeds, while the TC levels remained insignificantly altered, corroborating our experimental outcomes. However, Elnesr et al. (2019), observed a marked increase in serum TP upon the addition of sodium butyrate to the diet of Japanese quails, while ALB levels remained unaffected, which contrasts with our experimental results. These discrepancies may stem from the influence of different breeds, ages, genders, and dietary compositions. Ammonia is globally recognized as one of the pollutants (Li et al., 2023). The reduction in the concentration of AN in the organism reflects an enhancement in protein degradation metabolism (Tao et al., 2021). Our study results reveal a significant reduction in the serum AN levels upon the addition of CSB, echoing the changes observed in TP and ALB levels. These findings suggest that the inclusion of CSB in the diet effectively elevates the serum TP and ALB levels in yellow-feathered broiler chickens while concurrently reducing AN content and ameliorating protein breakdown metabolism in poultry.

The morphological structure of the small intestine is crucial for the digestion and absorption of nutrients in broiler chickens (Moreno-Mendoza et al., 2021). Increasing the mucosal surface area in the intestine can expedite the transfer of nutrients to the circulatory system (Taylor et al., 2021). A shallow crypt depth and tall villi height can augment the mucosal surface area in the intestine, serving to sustain normal intestinal development and enhance the rate of nutrient absorption (Paul et al., 2021). Zhang et al. (2022), confirmed that adding CSB to the diet of laying hens significantly increases the VH in the jejunum and ileum. Similarly, Liu et al. (2017), observed significant improvements in the VH, CD, and V/C ratio in the small intestine of broiler chickens upon the addition of CSB to their diets. Our findings align with previous results, indicating a significant enhancement in the intestinal morphology of yellow-feathered broiler chickens upon the addition of CSB. This study firmly establishes that the addition of CSB can ameliorate intestinal morphology, likely due to the slow release of butyric acid by CSB in the intestine, which provides ample energy substrates for the differentiation of small intestinal epithelial cells (Guo et al., 2022). Consequently, this promotes an increase in VH and the expansion of the absorptive surface area of the small intestine, which may explain the observed improvement in the growth performance of broiler chickens upon the addition of CSB.

The gut microbiota plays a crucial role in the physiological and health status of livestock and poultry, particularly in the maintenance of intestinal health (Martin-Gallausiaux et al., 2021). The structure and composition of gut microbiota are influenced by various factors, such as breed, environment, rearing practices, and feed type (Diaz Carrasco et al., 2019). Previous research has demonstrated the beneficial role of sodium butyrate in promoting the balance of gut microbiota (Zhou et al., 2017; Dou et al., 2022; You et al., 2023). However, there is limited information regarding the impact of CSB on the intestinal microbiota of broiler chickens. Our study observed a reduction in the diversity and richness of cecal microbiota following the addition of CSB, contrasting with the findings by Xiao et al. (2023), indicating the need for further experiments to validate these observations, possibly due to differences in breeds, feed types, or rearing practices. In addition, our study also revealed a significant increase at the phylum level of *Bacteroidetes* and its genus *Bacteroides* in the CSB group. *Bacteroidetes* plays a role in degrading complex carbohydrates and synthesizing propionate, while *Bacteroides* provides nutrition to other microbial communities, safeguards the host from intestinal pathogens, and degrades polysaccharides to produce butyrate salt (Louis et al., 2014; Chen et al., 2019; Zafar and Saier, 2021). These results align with findings by Deng et al. (2023), who reported a notable increase in the abundance of Bacteroidetes in the gut upon the addition of CSB. Our results also revealed a significant reduction in the abundance of *Proteobacteria*, *Deferribacteres*, and *Epsilonbacteraeota* in the CSB group. *Proteobacteria* is considered an ecological dysbiosis and potential diagnostic feature of disease risk (Chen et al., 2021), *Epsilonbacteraeota* comprises only a few genera, such as *Helicobacter* and *Campylobacter*, most of which are pathogenic, and *Deferribacteres* is associated with obesity (Walker et al., 2014). These changes suggest that the addition of CSB effectively improves the balance of gut microbiota, promoting the proliferation of beneficial bacteria and reducing the abundance of potentially harmful bacteria. Therefore, our study indicates that the appropriate addition of CSB to the diet of broiler chickens is beneficial for the cecal microbiota.

## Conclusion

Supplementing CSB in the diet increased the production performance of yellow-feathered broilers, enhanced their body’s antioxidant capacity and serum biochemical levels, improved intestinal morphology, and intestinal barrier function. Additionally, the addition of CSB reduced the abundance of harmful bacteria in the cecum, promoted the proliferation of beneficial bacteria, and had a significant improvement effect on intestinal microbiota. Therefore, these results indicate that the addition of CSB is beneficial for the healthy growth of yellow-feathered broilers.

## Data availability statement

The datasets presented in this study can be found in online repositories. The names of the repository/repositories and accession number(s) can be found at: https://ngdc.cncb.ac.cn, PRJCA022760.

## Ethics statement

The animal studies were approved by Animal care and use protocol was approved by the Institutional Animal Care and Use Committee of the Zhejiang Academy of Agricultural Sciences (approval number: 2022ZAASLA59), which was following the Guidelines for Experimental Animals established by the Ministry of Science and Technology (Beijing, China). The studies were conducted in accordance with the local legislation and institutional requirements. Written informed consent was obtained from the owners for the participation of their animals in this study.

## Author contributions

JH: Conceptualization, Software, Writing – original draft, Writing – review & editing. LLu: Data curation, Writing – review & editing. LLi: Data curation, Writing – original draft. YT: Methodology, Writing – review & editing. TZ: Methodology, Writing – review & editing. YM: Software, Writing – review & editing. SL: Software, Writing – review & editing. LC: Methodology, Writing – review & editing. WX: Methodology, Writing – review & editing. TG: Data curation, Writing – review & editing. GL: Data curation, Writing – review & editing. XL: Conceptualization, Writing – review & editing.

## Footnotes

^1^http://ccbjhu.edu/software/FLASH/

^2^http://greengenes.secondgenome.com/
